# High prevalence of abdominal aortic aneurysm in older men with cerebrovascular disease: Evaluation of a local screening program

**DOI:** 10.3389/fneur.2023.1131322

**Published:** 2023-04-11

**Authors:** M. Loban, J. W. C. Gratama, P. L. Klemm, Roeland B. Van Leeuwen, H. Vriesema, Henri Paul Bienfait

**Affiliations:** ^1^Department of Neurology, Gelre Hospital, Apeldoorn, Netherlands; ^2^Department of Radiology, Gelre Hospital, Apeldoorn, Netherlands; ^3^Department of Vascular Surgery, Gelre Hospital, Apeldoorn, Netherlands; ^4^Department of Biometrics, Gelre Hospital, Apeldoorn, Netherlands

**Keywords:** stroke, ischemic attack, transient, aortic aneurysm, abdominal, mass screening, prevalence

## Abstract

**Introduction:**

Patients with cerebrovascular disease may suffer from other vascular morbidities, such as abdominal aortic aneurysm (AAA). Previously, a high prevalence of AAA has been demonstrated in men 60 years of age and older who have experienced TIA or stroke. This report evaluates the results of a decade's operation of a local screening program for AAA in this selected neurologic population.

**Methods:**

Men aged ≥60 years and admitted to the neurology ward of a community-based hospital in the Netherlands from 2006 to 2017 with a diagnosis of TIA or stroke were selected for screening. The diameter of the abdominal aorta was assessed by abdominal ultrasonography. Patients with detected AAA were referred for evaluation by a vascular surgeon.

**Results:**

AAA was detected in 72 of 1,035 screened patients (6.9%). AAAs with a diameter of 3.0–3.9 cm accounted for 61.1% of the total aneurysms found; AAAs with a diameter of 4.0–5.4 cm accounted for 20.8% of the total; and large aneurysms with a diameter of ≥5.5 cm accounted for 18.1% of all aneurysms discovered. A total of 18 patients (1.7%) underwent elective aneurysm repair.

**Discussion:**

The detection rate of AAA in older men with cerebrovascular disease was roughly 5-fold the detection rate in known European screening programs in older men from the general population. The proportion of large AAAs (≥5.5 cm) was also substantially higher. These findings reveal a previously unknown co-morbidity in patients with cerebrovascular disease and may be helpful for cardiovascular management of this large group of neurologic patients. Current and future AAA screening programs may also benefit from this knowledge.

## Introduction

Cerebrovascular disease is one of the sequelae of systemic atherosclerotic vascular disease. Patients who experience transient ischemic attack (TIA) and stroke may suffer from other vascular morbidities, such as abdominal aortic aneurysm (AAA). AAA is a potentially fatal condition with a high rate of mortality when the aneurysm ruptures (1). Elective aneurysm repair reduces mortality, and its perioperative risks are decreasing (2). However, AAA is a silent disease, and most aneurysms are detected by chance on imaging studies conducted for other purposes. When AAA is detected, patients are often put under the surveillance of a vascular surgeon. Elective AAA repair is recommended for large AAAs (≥5.5 cm) when the rupture risk outweighs the surgery risks (3).

In 2009, our research group demonstrated high prevalence of AAA in TIA or stroke patients (4). The subpopulation with the highest risk of AAA was men aged ≥60 years, regardless of stroke type, TOAST classification, and presence of known cardiovascular risk factors. The results of the study were in line with large studies on the prevalence of AAA, indicating the highest prevalence in older men. However, the detection rate observed in our study, of 11% in older men who have experienced TIA or stroke, roughly twofold the prevalence observed in older men from the general population in known trials (4–7). In light of these results, a local screening program for AAA has been implemented focusing on older men who have experienced TIA or stroke.

Since the late 2000s, nationwide screening programs for AAA have gradually been implemented in England and Sweden, and partially implemented in the United States, to reduce AAA-related mortality in men above 65 years of age (8–11). In multiple countries, the effectiveness of screening for AAA has been or is still being investigated, but this has not resulted in the implementation of screening programs with broad coverage (11). In a recent report, the Dutch Health Council did not advise routine population screening for AAA in the Netherlands after an extensive evaluation of existing trials and screening programs implemented worldwide (12). Although reports evaluating the existing large screening programs in England and Sweden do show clear benefits in terms of AAA-related mortality and low costs per quality-adjusted life-year (QALY) gained, the true prevalence of AAA in the screened groups turned out to be lower than expected (13–16). Multiple recent reports show a decreasing prevalence of AAA (17–19). This may imply a reduction in effectiveness of the existing screening programs and raises the need for adjustment of current screening strategies. Selecting a subpopulation with a higher detection rate of AAA may increase screening efficiency.

In the current report, we present the results over the past decade of our local screening program for AAA in older men who have experienced TIAs and stroke, and outline its clinical consequences for this group of patients. In addition, screening program outcomes are discussed in light of the results of other known screening programs worldwide.

## Methods

This study was performed at a community-based teaching hospital in the Netherlands. Screening for AAA was implemented as part of the stroke workup for men aged ≥60 years admitted to the Department of Neurology with a diagnosis of TIA or stroke, in accordance with the findings of our previous study at the same hospital (4). Prospectively collected data on all patients admitted to the neurology department were used to identify patients who underwent this screening. Patient files were used to retrieving clinical data including sex, age, date of occurrence of the cerebrovascular event, type of cerebrovascular event, date of abdominal aorta measurement and maximal diameter of the abdominal aorta, and (if applicable) the results of surveillance by the vascular surgeon and the date and type of eventual surgery.

All men aged ≥60 years admitted to the Department of Neurology between January 2006 and December 2017 with a diagnosis of TIA or stroke were eligible for screening. The diagnosis was made by an attending neurologist. Exclusion criteria were subarachnoid hemorrhage; short life expectancy; expected poor functional outcome with expected discharge to a nursing home; clinical condition not allowing transportation within the hospital or making this undesirable; known AAA; previous abdominal aneurysm repair; and earlier radiological evaluation for AAA.

Screening was performed *via* ultrasonography of the abdominal aorta by an attending radiologist. AAA was defined as a maximal anterior–posterior diameter of the abdominal aorta of ≥3.0 cm. The ultrasonography was carried out in parallel to the usual stroke workup administered during the same admission. The in-room duration of the ultrasonography exam was under 5 min. Patients with a newly discovered AAA were referred to a vascular surgeon for surveillance and eventual elective repair of the AAA.

The Institutional Review Board of our institution approved the screening program.

## Results

From January 2006 to December 2017, 1,037 male patients aged ≥60 years and admitted to the Department of Neurology with a diagnosis of stroke or TIA were selected for AAA screening. The median age at the time of admission and screening was 73 (IQR: 67; 79). A total of 642 patients (61.9%) had a diagnosis of acute ischemic stroke, 40 (3.9%) had an intracerebral hematoma (ICH), and 355 (34.2%) were diagnosed with a transient ischemic attack (TIA). A reliable measurement of the diameter of the abdominal aorta was successfully obtained in 1,034 patients (99.7%).

Abdominal aortic aneurysm (anterior–posterior diameter ≥3.0 cm) was found in 72 patients (6.9%). In 44 patients (4.2%), the size of the AAA was between 3.0 and 3.9 cm; in 15 cases (1.4%), the diameter was between 4.0 and 5.4 cm; and in the remaining 13 patients (1.3%), the size of the aneurysm was equal to or exceeded 5.5 cm. Thus, AAAs with a diameter of 3.0–3.9 cm accounted for 61.1% of the total aneurysms discovered; those with a diameter of 4.0–5.4 cm accounted for 20.8% of the total; and large aneurysms with a diameter of ≥5.5 cm accounted for 18.1% of all those discovered. The median age of men with a newly discovered AAA was 77 (IQR: 69; 83) years.

A total of 26 patients did not undergo follow-up with a vascular surgeon: in 13 cases this was for unknown reasons/the patients were lost to follow-up; seven patients were not followed up due to deterioration of their clinical condition; one patient died before the follow-up appointment could take place; three declined follow-up; and two moved to another region. A total of 48 patients were evaluated by a vascular surgeon. As of August 2018, 28 of these were either known to be still under the surveillance of a vascular surgeon or surveillance had ended due to stability of the size of the AAA. One patient was indicated for aneurysm repair after 48 months of follow-up but declined surgery. A total of 18 patients (1.7%) underwent surgery: nine within a short period (1–15 weeks) after AAA was discovered and nine after a follow-up period with a median duration of 40 months (range: 7–128 months). Four patients underwent an open procedure, and 14 patients underwent endovascular aneurysm repair (EVAR). Two patients underwent re-operation due to major peri-operative complications, both after EVAR; two patients needed a second surgery due to technical failure of endovascular abdominal aortic aneurysm repair; one patient had a ruptured aortic aneurysm 4 years after initial surgery (EVAR) due to progression of the disease. No aneurysm-related or peri-operative deaths are known to have occurred in our patient population.

**Figure 1:**
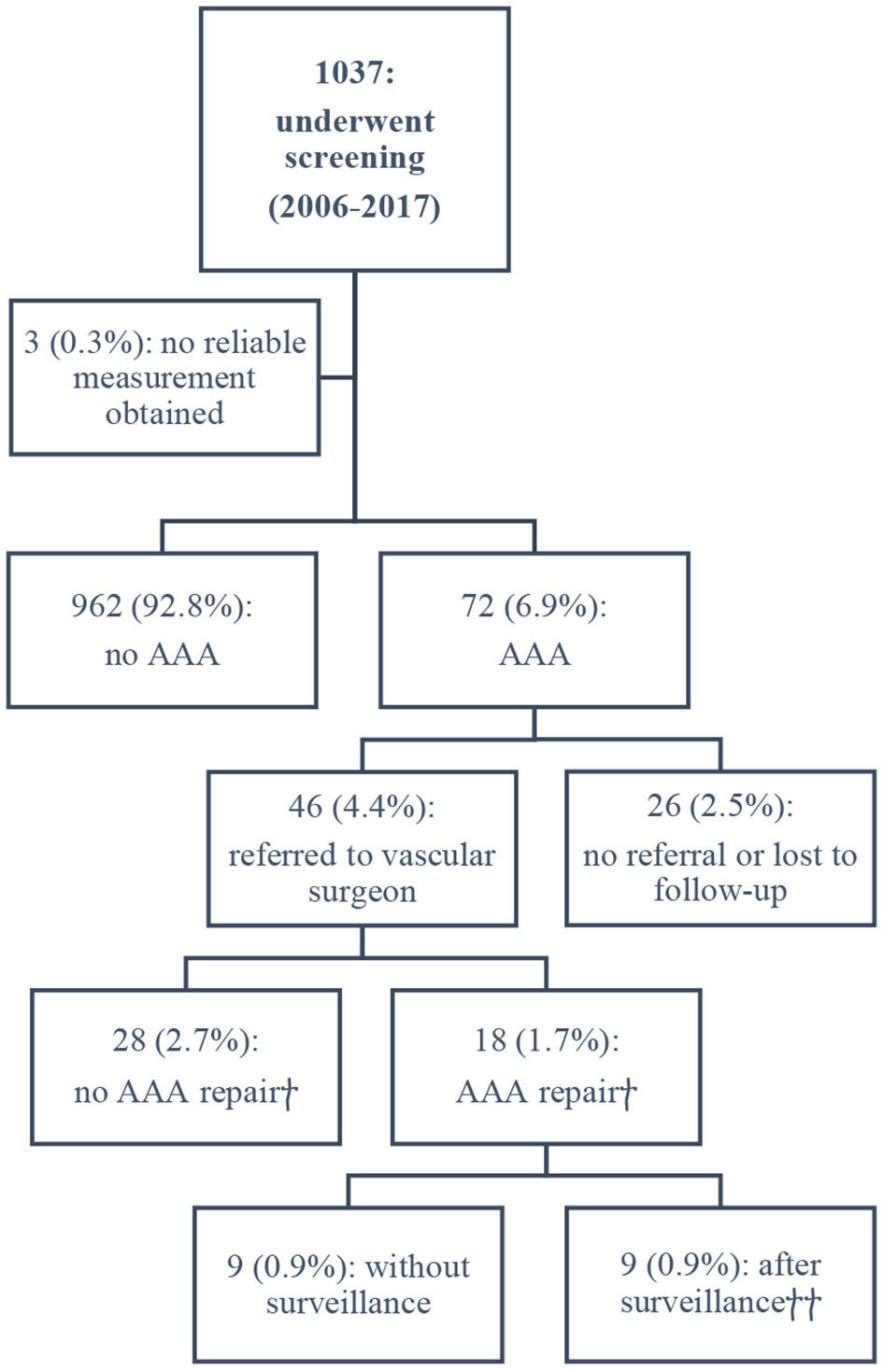
Outcomes of the screening program. ^†^Based on available data as of August 2018. ^††^Median surveillance period: 40 months.

The results of the screening program are summarized in Figure 1.

The actual screening rate among all men admitted with TIA or stroke and aged ≥60 years was ~45%. Due to the retrospective nature of the analysis, the data from the years 2006–2009 regarding the screening rate among eligible patients are incomplete; i.e., it is unknown how many patients did not undergo echographic aorta measurement between 2006 and 2009 due to exclusion criteria or logistical failures. During the years 2010–2017, 1,295 potentially eligible patients were evaluated at our stroke department. A total of 574 patients (44.3%) underwent screening and 721 (55.7%) did not. In 192 patients (14.8%), screening was not performed due to poor clinical condition or expected poor functional recovery; 72 patients (5.6%) had a known AAA; 132 (10.2%) had already participated in our screening program during a prior admission; 90 (7.0%) had undergone recent abdominal imaging without any signs of AAA; 13 (1.0%) did not give consent or were urgently transferred to another hospital; and in 222 cases (17.1%), no clear reason why screening was not performed could be identified. As mentioned above, the exact screening rate for the years 2006–2009 is unknown. However, there is no objective reason to assume that there was a substantial difference in the screening rate compared to the years 2010–2017.

## Discussion

This report evaluates the results of a local screening program for AAA over a period of 11 years. The screening program was implemented following a preliminary study detecting a group of patients at risk of AAA (men aged ≥60 years with a diagnosis of TIA or stroke). The program was designed, on the one hand, to improve the cardiovascular risk management of patients with known cerebrovascular morbidity; and, on the other hand, to maximize the detection of asymptomatic but potentially fatal conditions in the region (with a population of >200,000).

The screening rate in older men admitted with TIA or stroke was ~45%; 17% of potentially eligible patients were not screened due to logistic failures, and the remaining proportion of patients did not meet the inclusion criteria due to poor clinical condition or having previously undergone aorta imaging. It should be noted that 5.6% of patients were excluded from screening due to already-known AAA. The prevalence of asymptomatic AAA discovered among the screened patients was 6.9%, among which 18.1% of patients had large aneurysms with a diameter of ≥5.5 cm. The prevalence of AAA discovered was lower than the previously demonstrated prevalence of 11% in a comparable cohort at our institution. This decrease is in line with the results from other screening programs worldwide, showing a lower true prevalence of AAA than that identified in large RCTs.

Nevertheless, the detection rate of AAA in this screening setting, with a focus on a select group of patients with cerebrovascular disease, was substantially higher than the detection rate in other European screening programs with unselected men from the general population (Table 1). A recent report on the Veteran Affairs AAA screening program (USA) shows an AAA detection rate comparable to that of our cohort (20). It must be noted that a substantial proportion of the US cohort did not meet the initial age and gender inclusion criteria for screening; thus, the comparison with our Dutch cohort must be made with caution. Nevertheless, we do not have a clear explanation for the high detection rate in our study. Remarkably, older men with stroke or TIA in our cohort had at least a fivefold risk of having a large (≥5.5 cm) AAA at first ultrasonography compared to the risk observed in all other known screening programs. The proportion of patients with screening-detected AAAs who underwent surgical repair was also higher in our screening program, which cannot be attributed to more aggressive surgical management. However, there is heterogeneity in this comparison, and some data are unavailable in other reports. Nevertheless, the high detection rate of AAA demonstrated here, especially the high proportion of large AAAs in our cohort of older men with cerebrovascular disease, is remarkable when compared to programs involving screening of unselected older men from the general population (Table 1).

**Table 1:** Comparison of outcomes between known screening programs.

**Screening program**	**Screened group**	**Period of screening**	**Number of subjects**	**AAA detected**	**Large AAA (≥5.5 cm) at first scan**	**AAA repair surgery (including surgery after period of surveillance)**
ACVA (Netherlands; current report)	Men ≥ 60 y/o admitted with stroke or TIA	2006–2017	1,037	72 (6.9%)	13 (1.3%)	18 (1.7%)
NAAASP (England) (16)	One-time screening at age 65	2009–2013	700,000	9,388 (1.3%)	755 (0.1%)	870 (0.1%)
SASS (Sweden) (13)	One-time screening at age 65	2006–2014	253,896	3,891 (1.5%)	Appr. 272 (0.1%)^†^	Appr. 1,100 (0.4%)^†^
VANCHCS AAA screening (USA) (20)	Veterans 65–75 y/o who have smoked >100 cigarettes in their lifetime^††^	2007–2016	19,649	1,232 (6.3%)	44 (0.2%)	54 (0.3%)
UCC-SMART (Netherlands) (18)	Patients 40-80 y/o with a history of manifest atherosclerotic disease	1996−2018	5,540 (men)	136; men: 2.5%	6 (0.1%)	49 (0.9%)

† Only percentage available; approximate number calculated using published percentages.

†† Heterogeneous group, including 18.7% not meeting the age and/or gender inclusion criteria.

The high prevalence of AAA in our series of studies reveals previously unknown co-morbidity in patients with cerebrovascular disease. To the best of our knowledge, the prevalence of AAA in patients who have experienced TIA or stroke has not been investigated before by any other research group. However, the same association has been reported previously in reverse. A history of cerebrovascular disease is frequent (13.3–27.4%) in patients with known asymptomatic and acute AAA, as is a history of coronary artery disease (18.2–26.7%) (21–23). A history of vascular disease, including TIA or stroke, acute coronary disease, and peripheral artery disease, has been shown to be present in 57% of patients with acute AAA in a study of a series of such patients (21). Patients with known coronary artery disease are at a higher risk of AAA, and higher severity of coronary artery disease is associated with higher prevalence of AAA (24, 25). The prevalence of AAA was found to be 5.7% in a cohort of 438 men with coronary artery disease (24). Another study revealed a comparable prevalence (4.2%) among male patients undergoing coronary angiography, which increased drastically in patients aged ≥65 years (8.6%) and even further in patients with three-vessel coronary disease (14.4%) (25). Since cerebrovascular disease and coronary artery disease may share a common pathophysiological ground, the high prevalence of AAA in patients with coronary artery disease indirectly supports our findings.

## Limitations

A limitation of our study may be selection bias. Approximately 45% of eligible patients underwent screening; 38% of patients did not meet the screening criteria, and 17% did not undergo measurement of the aorta diameter for unidentifiable reasons (Figure 1). We assume that these cases were missed because of logistical failures. Another limitation is the generalizability of our findings to other populations. Screening was performed in a community-based hospital in an area with a predominantly Caucasian population, and there are no data regarding the vascular risk factors in the study population. However, the absence of data on such risk factors has a plausible explanation, since in our preliminary study, only male gender and age ≥60 years were found to correlate with higher prevalence of AAA, while other cardiovascular risk factors did not (4). Wide inclusion criteria facilitate rapid identification of patients eligible for screening, which is important in daily practice, especially in a non-academic setting. In this report, a comparison of AAA prevalence was made with the prevalence observed in other screening programs. This comparison has a drawback in the form of heterogenicity of the cohorts. The higher prevalence of AAA in our screening program as compared to other programs may be partly attributed to a slight difference in the age of patients. The median age at the time of screening in our study was 73 years, while in other European screening programs, subjects have received an invitation for screening at the age of 65. Based on the results of our research, we cannot estimate to what extent the older age of patients contributed to the higher rates of detection of AAA, as there was no control group of patients without a diagnosis of TIA or stroke.

## Conclusion

The high prevalence of AAA in general, and the larger proportion of large AAAs in older men with cerebrovascular disease, encourages the continuation of the established screening program, as it is likely that it leads to better detection and management of co-morbidity in this large subpopulation of neurologic patients. A screening program for AAA becomes beneficial in terms of cost-effectiveness and reduction of AAA-related mortality when it achieves a detection rate of at least 0.5% (26). It is reasonable to assume that this target for effectiveness has been reached in the case of the current screening program, with a detection rate of nearly 7%, especially when taking into consideration the high proportion of large AAAs with no need for follow-up. However, the life expectancy, and thus the probability of mortality as a result of a ruptured AAA, among patients who have experienced TIA or stroke may differ from that among men in the general population. This may decrease the benefit of the screening method presented. Predictors of mortality or dependency after TIA or stroke (27), such as stroke severity, recurrent stroke, increasing age, and vascular co-morbidity, should be taken into account when selecting men suitable for screening from a population with known cerebrovascular disease.

In conclusion, abdominal aortic aneurysm may be a frequent vascular co-morbidity of cerebrovascular disease, especially in men. This knowledge may be helpful for the cardiovascular management of stroke patients, as well as for the development of current and future population-wide screening programs for AAA (28–30). A further, more expansive review of the literature on this subject strengthens our recommendations (31–37). Screening of men aged ≥60 years with a diagnosis of TIA or stroke for this condition seems to be highly effective and is easy to carry out. Validation of these results in another cohort should be considered by neurological societies. Confirmation of high prevalence of AAA in a similar cohort could lead to new insight into optimal vascular risk management in a large group of neurologic patients.

## Data availability statement

The raw data supporting the conclusions of this article will be made available by the authors, without undue reservation.

## Ethics statement

Ethical review and approval was not required for the study on human participants in accordance with the local legislation and institutional requirements. Written informed consent from the patients/participants or patients/participants' legal guardian/next of kin was not required to participate in this study in accordance with the national legislation and the institutional requirements.

## Author contributions

ML and HB researched literature and conceived the study. HB was involved in gaining ethical approval. ML wrote the first draft of the manuscript. All authors reviewed and edited the manuscript and approved the final version of the manuscript.
